# Production of ultrasonic vocalizations by *Peromyscus *mice in the wild

**DOI:** 10.1186/1742-9994-3-3

**Published:** 2006-02-28

**Authors:** Matina C Kalcounis-Rueppell, Jackie D Metheny, Maarten J Vonhof

**Affiliations:** 1 Department of Biology, University of North Carolina at Greensboro, 1000 Spring Garden Street, Greensboro, North Carolina, USA; 2 Department of Biological Sciences, Western Michigan University, 1903 W. Michigan Avenue, Kalamazoo, Michigan, USA

## Abstract

**Background:**

There has been considerable research on rodent ultrasound in the laboratory and these sounds have been well quantified and characterized. Despite the value of research on ultrasound produced by mice in the lab, it is unclear if, and when, these sounds are produced in the wild, and how they function in natural habitats.

**Results:**

We have made the first recordings of ultrasonic vocalizations produced by two free-living species of mice in the genus *Peromyscus *(*P. californicus *and *P. boylii*) on long term study grids in California. Over 6 nights, we recorded 65 unique ultrasonic vocalization phrases from *Peromyscus*. The ultrasonic vocalizations we recorded represent 7 different motifs. Within each motif, there was considerable variation in the acoustic characteristics suggesting individual and contextual variation in the production of ultrasound by these species.

**Conclusion:**

The discovery of the production of ultrasonic vocalizations by *Peromyscus *in the wild highlights an underappreciated component in the behavior of these model organisms. The ability to examine the production of ultrasonic vocalizations in the wild offers excellent opportunities to test hypotheses regarding the function of ultrasound produced by rodents in a natural context.

## Background

Ultrasound is commonly used for orientation and prey localization by diverse taxa, including bats, odontocete whales, insectivores, and rodents. Ultrasonic signals in these groups range from simple broadband clicks produced by whales, insectivores, and some megachiropteran bats, to highly modified, tonal signals that show structured change over time as in microchiropteran bats [1]. However, in addition to their function in orientation, these signals may also have social functions, including communication of individual identity or group membership, kin recognition, alarm communication, information transfer, infant-mother communication, mate attraction, and territorial defence [2-5].

In contrast to our wealth of knowledge on the use of ultrasound by microchiropteran bats and odontocete whales, we know comparatively little about the use of ultrasound by rodents in the wild [but see [5]]. However, there has been extensive research on rodent ultrasound in the laboratory, where ultrasonic vocalizations (henceforth USVs) have been documented for a number of rodent species, particularly within the superfamily Muroidea [6,7]. A major impetus for this large body of research is that the two major mammalian non-human models are muroids: the lab mouse (*Mus musculus*) and lab rat (*Rattus norvegicus*). Mice and rats have historically been used for classical human biomedical research and, more recently with the sequencing of both genomes [8,9], have become the main models for the basis of human and mammalian development and behavior [10,11]. Because both rat and mouse infants predictably produce USVs in the laboratory, their USVs are regularly used as phenotypic markers in neurobehavioral development [12]. The study of USV production by rats and mice in the laboratory has become so prevalent that a recent effort was made to standardize methods in the study of laboratory rodent USVs [13]. In addition, a detailed study of USVs within individual male mice revealed variation in syllable usage and timing, indicating that laboratory mice are capable of producing song [14].

Although the majority of research on USVs has occurred in only two model muroid rodent species, the Muroidea is the largest and most diverse superfamily of mammals with over 1300 species in 5 families [15,16]. Based on laboratory research, it appears that USV production may be common within the Muroidea. Within the muroids, USVs have been documented in 18 genera (B. H. Blake, unpublished data) in the subfamilies Arvicolinae, Cricetinae, Gerbillinae, Murinae, and in the Neotominae-Sigmodontinae [17-22]. Muroids examined in the lab have been shown to produce USVs as juveniles, adults or both, but the context of USV production varies [6,23]. Neonates and juveniles produce USVs in response to isolation from parents, cool temperatures, handling, anticipation of play, or painful stimuli [6,17,24-34]. Adult males and/or females produce USVs during agonistic (mostly intrasexual) interactions [35,36] and during courtship and mating [6,37,38]. Adult males additionally produce ultrasonic songs when stimulated by conspecific urine [14], and adult females also produce ultrasound when their pups are removed from their nest [6].

Despite the valuable and extensive research on USVs in rodents in the lab, it is unclear if and when, these USVs are produced in the wild, and how they function in natural habitats. The exception to this is the recent discovery of the use of USVs by the Richardson's ground squirrel (*Spermophilus richardsonii*) to warn conspecifics of imminent danger [5]. To our knowledge, this is the only test of the functional significance of USV production by rodents in the wild. Without understanding the context of USV production in the wild, it is difficult to understand the selective pressures leading to their evolution and maintenance. Furthermore, although it may be possible to attribute a function to the USVs produced in the lab, it is critical to understand their adaptive significance in a natural context. Understanding the context and function of USV production by wild muroid rodents is especially relevant given the prevalence of behavioral research related to USV production by lab rats and mice. It has been suggested recently that inbreeding of laboratory mice may have acted to reduce the variation in the USVs produced by laboratory mice, and that wild mice might exhibit higher diversity and complexity of USVs and ought to be used for comparisons of song production with other animals [14].

The purpose of our study was to passively record and characterize USVs given by free-living muroid rodents in the wild. Here we document and characterize, for the first time, USVs given by wild *Peromyscus *mice in the wild. We examined USV production in two syntopic species of *Peromyscus *(*P. californicus *and *P. boylii*) on long term study grids at the Hastings Natural History Reserve (HNHR), Monterey Co., California. Mice in the genus *Peromyscus *are models in both field and laboratory research relating to questions of mammalian evolution [39-41], ecology [42-44], and behavior [45-47]. We chose these two particular species of *Peromyscus *because long term studies of wild populations afforded us detailed knowledge of their ecology and behavior. In addition, time spent in the field has provided us with anecdotal observations of vocalizations being produced by these species.

## Results

Our recordings were made during the breeding season for both *P. boylii *and *P. californicus*. For both species, females were lactating and males had descended testes. Juveniles of both species were captured at adjacent trap stations indicating the emergence of litters. Many individuals that we captured were residents that had been ear-tagged in the previous breeding season.

**Figure 1:**
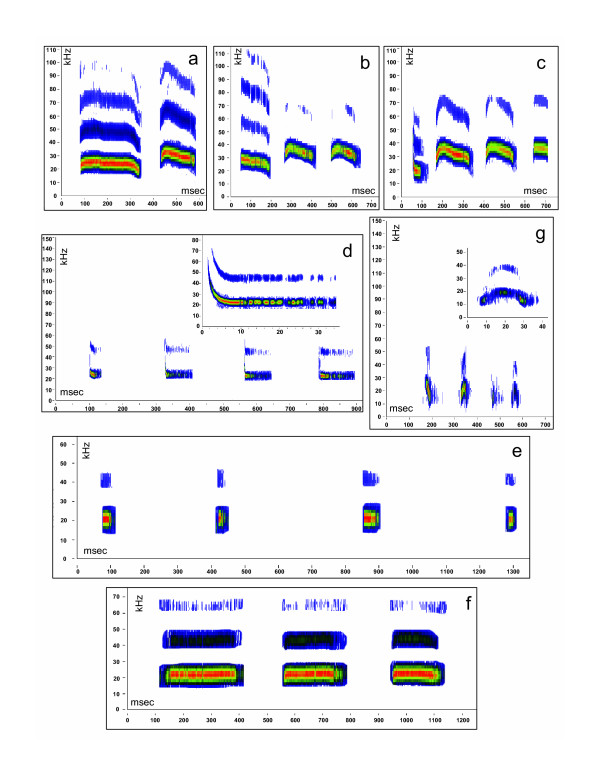
**Spectrograms of the seven *Peromyscus *motifs**. Characteristic spectrograms [frequency (kHz) vs. time (ms) graphs] of the 7 motifs. Amplitude levels are denoted by color with the highest being red and the lowest being blue. All panels reflect whole phrases recorded; a) two part whistle (2PW), b) three part whistle (3PW), c) four part whistle (4PW), d) frequency modulated short 20 (FMS20), e) short 20 (S20), f) long 20 (L20), g) BARK. The insets on the frequency modulated short 20 and the bark motifs are single syllables from the spectrogram, expanded on the x-axis, to show defining detail. For quantitative details of syllables, see text and descriptive statistics in additional file 10. For this figure, background noise has been removed from spectrograms to enhance the clarity of the syllables. To hear original recordings of each panel, listen to additional files 1 through 7.

On 6 nights of recording, we recorded a total of 65 USV phrases corresponding to 7 different motifs (Figure 1; Table 1). The 65 USV phrases were not evenly distributed among the 6 nights (mean ± 1SE = 10.8 ± 3.8, range = 2–29; χ^2 ^= 40.2, df = 5, p < 0.0001). We never recorded a single syllable; there were always at least two syllables recorded. Individuals never produced consecutive phrases; in a recording from a given individual there was always only one phrase (see Figure 1). However in two instances, we recorded consecutive phrases from different animals (based on clear intensity differences between the phrases) in the same recording. No motifs were only recorded at a single recording station (Table 1). Some motifs were recorded on both focal areas (Upper and Middle; see Methods and Table 1). Only one identical phrase was recorded simultaneously at more than one station, suggesting that the majority of USVs were not detectable greater than 5 meters away using our recording system.

**Table 1:** Type and number of motifs recorded on Upper and Middle sections of LRC grid.

	**Upper LRC Grid**									
	**v**	**w**	**u**	**s**	**p**	**o**	**r**	**t**	**q**	**Total**
**2 part whistle (2PW)**	1				1				1	**3**

**3 part whistle (3PW)**					3	1	1	1	10	**16**

**4 part whistle (4PW)**				1					10	**11**

**Frequency Modulated Short 20 (FMS20)**		1	1			2		1		**5**

**Short 20 (S20)**	2	1		2	1				2	**8**

**Long 20 (L20)**					1					**1**

**BARK**									3	**3**

**Total**	**3**	**2**	**1**	**3**	**6**	**3**	**1**	**2**	**26**	**47**
	**Middle LRC Grid**									
	**b**	**d**	**e**	**g**	**h**	**i**	**j**	**l**	**m**	

**2 part whistle (2PW)**		1		1		1	1	1		**5**

**3 part whistle (3PW)**			2							**2**

**4 part whistle (4PW)**	1			1						**2**

**Frequency Modulated Short 20 (FMS20)**										

**Short 20 (S20)**										

**Long 20 (L20)**				5	3					**8**

**BARK**									1	**1**

**Total**	**1**	**1**	**2**	**7**	**3**	**1**	**1**	**1**	**1**	**18**

Column headings refer to recording stations. Refer to text and Figure 4 for microphone locations. Row headings refer to motif. Totals are at the end of each row and column. Grand total is 65.

### Description of recorded ultrasonic vocalizations

The seven motifs were all easily distinguished from one another (Figure 1). Acoustic parameters of the seven motifs upon which the following details are based can be found in additional file 10. Because all phrases clearly fall into one of the following 7 motifs, and because our sample size within motifs is small, we do not describe syllable types in detail here but rather concentrate on overall motif characteristics. The "two part whistle motif" (henceforth 2PW) consisted of two ultrasonic long (each >100 ms) syllables (mean F max of each ~25 kHz) separated by an interval between syllables of approximately 90 ms (Figure 1; listen to additional file 1). The "three part whistle" motif (henceforth 3PW) consisted of three ultrasonic long (each >100 ms) syllables (mean F max ~21, ~24, ~26 kHz, respectively) separated by intervals between syllables of approximately 125 ms (Figure 1; listen to additional file 2). The "four part whistle" motif (henceforth 4PW) consisted of four ultrasonic long (ranging from 65–180 ms) syllables (mean F max ~20, ~25, ~25, ~27 kHz, respectively) separated by intervals between syllables of approximately 125–150 ms (Figure 1; listen to additional file 3). The 2PW, 3PW, and 4PW motifs all consisted of phrases with different syllable types (Figure 1). The 2PW, 3PW, and 4PW motifs were not simply variations of the same phrase with a varying number of syllables (i.e., the 2PW is not a 3PW or a 4PW cut off prematurely in recording). To demonstrate this, we analyzed all syllables within each motif (see data in additional file 10) and compared the first syllables among the 2PW, 3PW, and 4PW motifs. We found a significant difference among the motifs with respect to duration (ANOVA: F_2,36 _= 32.39, P < 0.0001) and ending F (ANOVA: F_2,36 _= 3.46, P < 0.05) of the first syllable. The first syllable of the 4PW motif was shorter in duration than the first syllable in both the 2PW or 3PW motifs (Tukey's *post hoc *p < 0.001) and the first syllable of the 4PW motif ended at a lower F than that of the 2PW motif (Tukey's *post hoc *p < 0.05; Figure 1; additional file 10).

The "short 20" (henceforth S20) and "frequency modulated short 20" (henceforth FMS20) motifs were similar to one another and consisted of short duration (<50 ms) syllables at 20–24 kHz separated by relatively long intervals between syllables (>300 ms; Figure 1). Unlike the 2PW, 3PW, and 4PW motifs, the syllable types within these motifs were the same. The main difference between the FMS20 and S20 motifs was that the syllables within the FMS20 (listen to additional file 4) phrases all began with a steep frequency modulated component to the syllable that resulted in a significantly higher starting F (ANOVA: F_1,11 _= 5.18, P < 0.05), larger band width (ANOVA:*F*_1,11 _= 5.01, P < 0.05), and larger slope (ANOVA:*F*_1,11 _= 5.95, P < 0.05) relative to the syllables of the S20 motif (listen to additional file 5).

Similar to the S20 motif, the "long 20" motif (henceforth L20) had repeats of the same syllable type and the syllable's fundamental frequency was at approximately 20 kHz, but the syllables were longer (~150 ms; ANOVA: F_1,15 _= 39.19, P < 0.0001; Figure 1; listen to additional file 6). The "bark" motif (henceforth BARK) consisted of very short (~20 ms) syllables that began and ended in the audible frequency range (12 kHz) but peaked at approximately 20 kHz with the interval between syllables being approximately 150 ms (Figure 1; listen to additional file 7). Syllables within all motifs had clear harmonic frequency components (Figure 1). There was considerable variation in the acoustic characteristics of 2PW, 3PW, 4PW, and BARK motifs (see descriptive statistics in additional file 10).

### Who is producing these ultrasonic vocalizations?

With the exception of a single pocket mouse (*Chaetodipus californicus*; captured on Middle), the only nocturnal rodent species resident on Upper and Middle during our recordings were *P. boylii *and *P. californicus*. There was a single woodrat (*Neotoma macrotis*) resident 10 m away from the NW edge of Upper, however we intensively recorded around the woodrat nest for 3 nights with 3 recording units and did not record any USVs. Other potential animals present that could produce ultrasound that our microphones would have recorded are insects, insectivores, and bats. These recordings are not insects because of the distinct lack of stridulation. We captured a single shrew (*Sorex trowbridgei*), the only insectivore present, in a Fitch trap along the bank of Upper, where we had recorded shrew echolocation (listen to additional file 8). We also recorded bat echolocation (listen to additional file 9) on both Upper and Middle. Both shrew [48,49] and bat [1] echolocation calls are markedly characteristic and different from the syllables and resultant motifs we present herein. Thus, the only nocturnal species that 1) were present on Upper and Middle, and 2) capable of producing these distinct ultrasound motifs were *P. boylii *and *P. californicus*.

**Figure 2:**
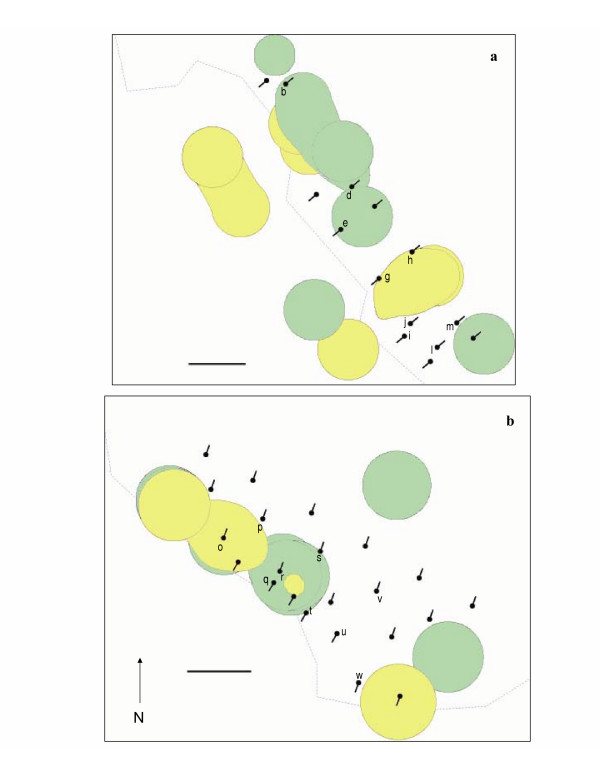
**Home ranges of individual *Peromyscus *in relation to microphones**. Kernel home range estimates (50%) for mice captured during study on a) Middle and b) Upper sections of the Lower Robertson Creek (LRC) Grid at HNHR. Creek denoted by dashed line. Microphones and the direction they were facing are indicated by directional microphone symbols. Only microphone stations that recorded USVs are labelled with letters (see Table 1). *P. boylii *ranges are green. *P. californicus *ranges are yellow. Horizontal line is 10 meters.

Our home range analyses indicated that both *P. boylii *and *P. californicus *were likely to have produced USVs (Figure 2). Based on home range data from Middle, there was one recording station (b) that recorded USVs (4PW motif) that had both *Peromyscus *species present. The recording stations that had only *P. boylii *present (d, e, m) recorded 2PW, 3PW, and BARK motifs. The recording stations that had only *P. californicus *present (g, h) recorded 2PW, 4PW, and L20 motifs (Figure 2; Table 1). On Upper, the majority of recording stations that recorded USVs (s, p, o, r, t, q) had both *Peromyscus *species present, and we recorded all seven motifs at these stations. We recorded S20 and FMS20 motifs at the only station (w) on Upper where only a single species (*P. californicus*) was present (Figure 2; Table 1). Based on these results both species produced USVs: at a minimum *P. boylii *produced 2PW, 3PW, and BARK motifs and *P. californicus *produced 2PW, 4PW, S20, FMS20, and L20 motifs.

## Discussion

For the first time, we have recorded ultrasonic vocalizations produced by two species of nocturnal *Peromyscus *in the wild. Our detailed knowledge of the habitat, community structure, and behaviors of these two species afforded us the opportunity to place a dense array of ultrasonic microphones in areas where only these species were present, and where these species exhibited exclusive home ranges. Our results, based on the location of recorded USVs and the home ranges of locally resident individuals, show that *Peromyscus *produce ultrasound in the wild. Furthermore, based on the exclusive (no overlap between species) home ranges of each species on Upper and Middle, we found that at the very least, *P. boylii *produces 2PW, 3PW, and BARK motifs and *P. californicus *produces 2PW, 4PW, L20, S20 and FMS20 motifs. It is probable that both species are capable of producing all motifs but this remains to be investigated.

In addition to demonstrating the production of USVs by *Peromyscus *in the wild, our results show that USV production is a common feature of the behavior of these nocturnal rodents and that there is variation in the types of USVs produced. On only six nights of recording, we recorded 65 individual high quality phrases and seven unique motifs. Furthermore, we found considerable variation in the acoustic characteristics of the syllables and phrases for all motifs, especially the 2PW, 3PW, 4PW, and BARK motifs, suggesting individual or contextual variation in the production of ultrasound.

The seven motifs superficially resemble laboratory recordings of rodent USVs [reviewed by [6,23]], but differ in frequency, duration, and harmonic content, which may or may not reflect differences in context and/or function. For example, the shape of the BARK syllables resemble frequency modulated syllables during mating behavior in *Apodemus *[23]; however the frequency of the BARK syllable is much lower (*Peromyscus *peak of syllable at approximately 23 kHz *vs *90 kHz for *Apodemus*). Likewise, the L20 syllable has a frequency that is similar to adult male rat submissive syllables (approximately 21 kHz *vs*. 25 kHz;[23]). The S20 syllable superficially resembles aggressive syllables produced by male rats in shape and duration, but is much lower in frequency (approximately 20 kHz *vs*. 50 kHz). We are unaware of any USVs recorded in the lab that resemble the first syllable of the FMS20 motif. The step-like frequency change within 2PW, 3PW, and 4PW motifs is reminiscent of the pattern seen in phrases emitted by infant *Microtus *[23]. These superficial similarities may reflect shared context and/or functions between behaviors of laboratory and wild mice. However further study is necessary, in part, because it is possible that similarities and/or differences may reflect the use of inbred laboratory strains in past studies. The temporal (seasonal) and demographic context as well as the function of the various (at least seven) motifs we have reported from the wild, remains to be determined.

Based on existing literature regarding USV production by muroid rodents in the laboratory and the functions of USVs and sonic vocalizations in other mammals and birds, USVs may function in a diversity of non-mutually exclusive contexts, including echolocation, offspring-parent communication, pair-bond maintenance, territorial defence, and mate attraction [1,6,23,28,30]. An alarm calling function [as in [5]] seems unlikely as alarm calling in rodents is associated with diurnality and sociality [50], neither of which are characteristic of the *Peromyscus *at HNHR.

In a recent study, Holy and Guo [14] argued that ultrasonic vocalizations produced by male laboratory mice in response to conspecific urine should be classified as songs. They based this classification on the following two characteristics of USV production by their mice. First, the USVs contained multiple syllable types, and second, the syllables are repeated in a regular manner over time. The 2PW, 3PW, and 4PW motifs we recorded clearly consist of multiple syllable types. However, we do not know if, or how often, 2PW, 3PW, and 4PW phrases are repeated by the same individuals. Characterizing 2PW, 3PW, and 4PW motifs as songs will only be possible with real time, continuous recordings of USVs and determination of the context and functions of these USVs.

## Conclusion

We have described the first recordings of ultrasonic vocalizations produced by two free-living species of mice in the genus *Peromyscus *(*P. californicus *and *P. boylii*) in California. The ultrasonic vocalizations we recorded are frequently produced and represent 7 different motifs. Within each motif, there was considerable variation in the acoustic characteristics suggesting individual and contextual variation in the production of ultrasound by these species. It remains to be seen if other species of wild *Peromyscus *or muroid rodents produce USVs, but given the breadth of documented production of USVs in the laboratory, it seems likely. The production of ultrasonic vocalizations by *Peromyscus *in the wild highlights an underappreciated component in the behavior of these model organisms and promises to be an exciting area of research in the fields of animal behavior, behavioral ecology, and sensory biology. Specifically, the variation in mating system between syntopic *P. californicus *and *P. boylii *offers excellent opportunities to test hypotheses regarding the function of USVs in *Peromyscus*, especially within the contexts of parental care, mate choice, and territorial defence. In general, the ability to examine the production of ultrasonic vocalizations in the wild offers excellent opportunities to test hypotheses regarding the function of ultrasound produced by rodents in a natural context.

## Methods

### Study species

Both *P. californicus *and *P. boylii *are sexually monomorphic and have similar schedules of gestation and lactation, diet, nest habitat, foraging habitat, and nightly activity schedules [51,52]. Based on data from the Hastings Natural History Reservation (HNHR), *P. californicus*, is exclusively monogamous [53] with a male/female pair nesting together during breeding and non-breeding seasons in an exclusive home range [54]. At HNHR, *P. boylii *shows variation in breeding system from polygyny to promiscuity. Males and females do not share nests, do not maintain long-term pair-bonds, and some litters are sired by more than one male [55]. At moderate (20 mice/ha) and high population densities (40–60 mice/ha) neither males nor females defend territories [55]. The home ranges of *P. californicus *pairs are exclusive of one another, while the home ranges of *P. boylii *have extensive intraspecific overlap. Thus, *P. californicus *and *P. boylii *are related species that live in the same habitat, have similar diets and life histories with respect to litter size and schedules of gestation and lactation, but differ with respect to mating system and territoriality.

### Study area and live trapping

**Figure 3:**
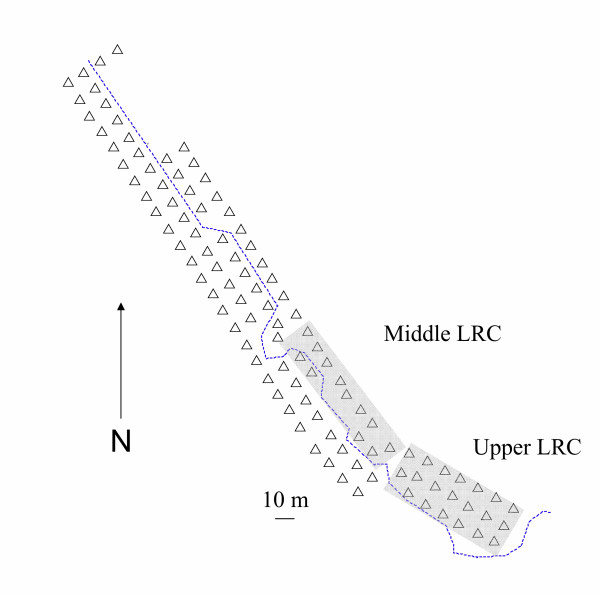
**Description of the LRC trapping grid**. Lower Robertson Creek (LRC) Grid at HNHR. Long-term trap stations [Δ]. Creek denoted by dashed line. "Middle" and "Upper" sections shaded. Horizontal line is 10 meters.

The Hastings Natural History Reservation (HNHR) is located in the foothills of the Santa Lucia mountains in upper Carmel Valley, California (Monterey County: 22.5 km SE Carmel Valley, 36°22'N, 121°33'W). Mean annual rainfall is 53 cm, occurring between November and April which corresponds to the breeding season of local *Peromyscus *species [56]. The HNHR encompasses three narrow valleys with habitat types including riparian, oak-bay woodland, chaparral, and grassland [57]. At HNHR *P. californicus *and *P. boylii *are syntopic and they are distributed in the dense understory of canyon bottoms of north-facing slopes [52,54]. In this habitat, there is a long-term live-trapping grid with approximately 10 m spacing that was established for the study of *P. californicus *and *P. boylii *(Lower Robertson Creek-"LRC"; Figure 3). A creek runs through the grid and both species prefer the live-oak (*Quercus agrifolia*) woodland riparian habitat that flanks the creek (approximately 20–30 m on each side). We have a very clear understanding of the community structure of small mammals on the LRC grid because it has been actively live-trapped for at least part of the year for most years from 1988–2005 and through the breeding season for nine of these years. On the LRC grid *P. californicus *and *P. boylii *live at moderate to high population densities (during acorn mast years approximately 20 individuals/ha and 20–60 individuals/ha, respectively). Other rodents on this grid live at relatively low densities (*Neotoma macrotis*, 5–10 individuals/ha; *Reithrodontomys megalotis*, *Chaetodipus californicus*, *Microtus californicus*, 1–2 individuals/ha). The only regular insectivore present is *Sorex trowbridgei *(1–2 individuals/ha).

### Determining home ranges of mice

By continuously live-trapping the long-term LRC grid, it was possible to determine the phenology and residency of individual mice. We trapped on both Upper LRC and Middle LRC (Figure 3; herein referred to as "Middle" and "Upper") from 6 Dec 2004 to 7 Jan 2005 to establish home ranges of individual residents. Minimally, one Sherman trap was used at each trap station. All traps were set 2–3 times weekly. Traps were baited with rolled oats, and standard mark and recapture techniques were used to determine sex, age, and reproductive condition of individuals [see [52]].

**Figure 4:**
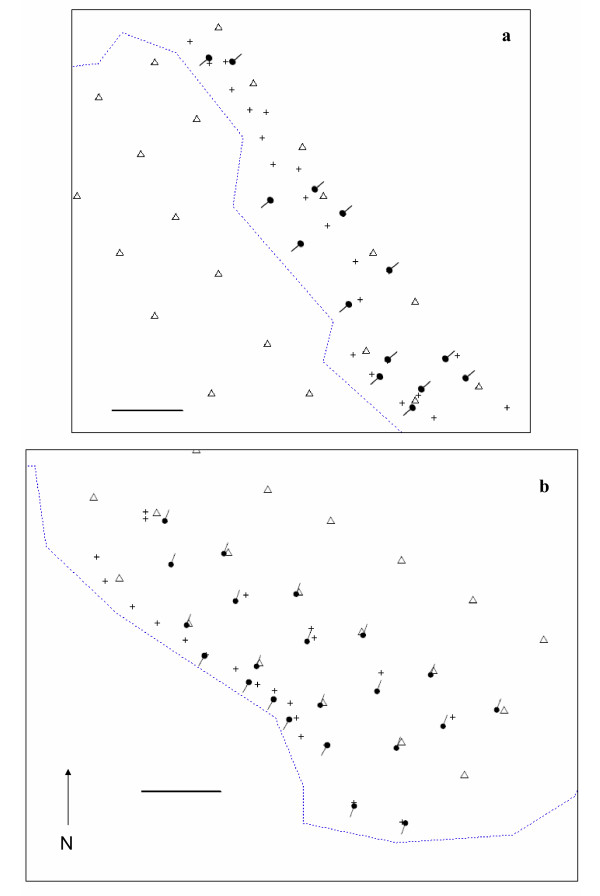
**Setup of microphones on Upper and Middle section of LRC trapping grid**. Set up for recording USVs on a) Middle and b) Upper sections of the Lower Robertson Creek Grid at HNHR. Regular trap stations [Δ]. Intensive trap stations [+]. Microphones and the direction they were facing are indicated by directional microphone symbols. Creek denoted by dashed line. Horizontal line is 10 meters.

Based on trapping data, Kernel home range estimates were calculated using ArcView 3.2 and Animal Movement SA v. 2.04 using all individuals we captured over the course of the study from both regular and intensive trap stations (see USV recording details and Figure 4). Although using data collected through trapping likely underestimates the size of home ranges of rodents [58], there is concordance between size and shape of ranges [59] calculated through trapping and radio-telemetry data. Kernel estimates were based on 3–6 trapping events using a smoothing factor of 5 with 50 % core ranges presented. If there were less than 3 trapping events for an individual, the individual's presence was indicated with a circular area the average size of the 50 % core range of conspecifics centred at the point of capture.

### Recording USVs

We conducted the recordings of USVs during the 2004/2005 breeding season after the establishment of residency for both species, from 12–18 Dec 2004. To record mouse USVs we established a grid of microphones (bat detectors) capable of recording broadband (10–120 kHz) ultrasound. We recorded with Pettersson D240x ultrasound detectors (Pettersson Elektronik AB, Uppsala, Sweden). These sampled at 307 kHz with 8 bit resolution. The bat detector was set to continuously record a 1.7 s loop of sound coming through the microphone. Upon detecting any sound in the range of 10–120 kHz the playback would be triggered and the previous 1.7 seconds of recorded sound would be played back, with time expanded by a factor of ten, into a voice activated tape recorder (Panasonic Mini Cassette Recorder RQ-L31) onto a low noise/high output audio cassette (Maxell P/I Communicator Series™ C120). The audio cassette recordings were played back in real time to a computer and saved as .wav files using SonoBat (DNDesign, Arcata, CA) directly onto the onboard computer sound card (Sigma Tel C-Major Audio) and resampled at 44.1 kHz with 16 bit resolution to retain the full signal quality of the original signal. We extracted time, amplitude, and frequency characteristics from sonograms rendered by SonoBat which used 1024 point fast Fourier transforms, 192 point windows, and varied window overlap so as to always render the sonogram with resolution greater than the screen pixel resolution. Our recording system had a frequency response up to the 12 kHz necessary to capture ultrasound up to 120 kHz (with the time expansion factor of ten). The maximum frequency resolution of the spectrographic analysis was 154 kHz.

We recorded for 3 consecutive nights separately on both Upper and Middle at 22 and 14 recording stations, respectively (Figure 4). From 12–14 December on Upper, the recording stations were placed approximately 5 m apart (Figure 4). The 5 m spacing was chosen to maximize the number of individual *Peromyscus *home ranges that we would be covering while also maximizing the probability of recording low intensity USVs. From 16–18 December on Middle, the recording stations were placed approximately 5–20 m apart and the spacing was chosen primarily based on suitable habitat availability (Figure 4).

At each recording station there were identical recording systems that consisted of one bat detector (microphone), acoustic cable, voice activated tape recorder and audio cassette. To protect against humidity and rain, the recording system was housed in two GladWare^® ^containers; one for the bat detector and one for the tape recorder. Two 15 mm holes were made in each container to accommodate the acoustic cable and a second 10 mm hole was made in the container with the bat detector to expose the microphone. The recording system was further housed within an open ended rectangular (38 cm width × 38 cm length × 10 cm height) plywood box to protect against wind and mechanical disturbance. The microphone on the detector was set horizontally approximately 25 cm above the ground facing out and flush with the opening of the box. Microphone direction was toward the water for the stations on top of the creek bank and toward the bank for the stations that were directly flanking the water (Figure 4), as individuals of both species tend to have high levels of activity near the bank edge [52].

Recording systems were set at sunset and retrieved the following morning. We did not live-trap while recording USVs to avoid influencing the results of acoustic monitoring. However, we saturated the area with additional Sherman traps (the intensive trap stations shown on Figure 4) and trapped the 3 nights following the last night of recording to confirm the presence and location of individual mice. In addition, we used 8 Fitch traps along the creek bank, near recording units to determine the presence of the local shrew species.

Any detected signals were digitized and analyzed using the sound analysis software program SonoBat the morning following the recordings. We analyzed syllables (see Vocalization Terminology below) using standard acoustic parameters including duration, starting frequency (henceforth F), ending F, high F, low F, bandwidth, F at maximum amplitude, slope, and duration between syllables.

All of these methods were conducted under an approved protocol of the University of North Carolina at Greensboro's Institutional Animal Care and Use Committee (UNCG-IACUC#05–08) and a scientific collecting permit from the California Department of Fish and Game (SCP#802046-01).

### Vocalization terminology

For clarity and comparison, we use the terminology of Holy and Guo [14]. A "syllable" is defined as a single discrete sound (separated by silence from other sounds). A "phrase" is defined as a succession of syllables where the time between syllables is less than 400 msec. A "syllable type" is a unique syllable that is recognizable and repeated either within or between phrases. Syllable types can differ in any of the following parameters: duration, low frequency (henceforth F), high F, starting F, ending F, F at maximum amplitude (henceforth Fmax), slope, and bandwidth. Each phrase is grouped according to syllable types, the number of syllables in a phrase, and the duration of time between syllables within a phrase. A "motif" is a sequence of syllables that were recorded repeatedly over time and that were statistically predictable based on characteristics of the syllables, the number of syllables in a phrase, and the duration of time between syllables within a phrase. All of our phrases fell into one of seven motifs, therefore the term "phrase" and "motif" can be used interchangeably. In general, we use "motif" when we are referring to the entire group of phrases, and phrase when we are referring to a particular sequence of syllables. Our USVs consisted of fundamental and harmonic frequencies. Throughout the paper, we characterize and discuss only the fundamental frequency of the syllable.

## List of abbreviations

"USV" Ultrasonic vocalization; "HNHR" Hastings Natural History Reserve; "LRC" Lower Robertson Creek; "F" frequency; "Fmax" frequency at maximum amplitude; "2PW" two part whistle; "3PW" three part whistle; "4PW" four part whistle; "FMS20" frequency modulated short 20; "S20" short 20; "L20" long 20

## Competing interests

The author(s) declare that they have no competing interests.

## Authors' contributions

MCKR and MJV, with equal contributions, conceived the ideas and developed this project. MCKR, MJV, and JDM participated in all aspects of field work and USV downloading and digitization in the field. JDM managed field work and provided significant input to the field method development. MCKR performed the analysis of acoustic characters, home range size, and statistical analyses. MCKR wrote the initial draft of the manuscript. Subsequent drafts were developed with continued and significant input from MJV. All authors read and approved the final manuscript.

## Supplementary Material

Additional File 1 Playback of the 2PW motif in Figure 1a with the time scale expanded by a factor of 10 to render the fundamental frequency audible to humans. Click here for file

Additional File 2 Playback of the 3PW motif in Figure 1b with the time scale expanded by a factor of 10 to render the fundamental frequency audible to humans. Click here for file

Additional File 3 Playback of the 4PW motif in Figure 1c with the time scale expanded by a factor of 10 to render the fundamental frequency audible to humans. Click here for file

Additional File 4 Playback of the FMS20 motif in Figure 1d with the time scale expanded by a factor of 10 to render the fundamental frequency audible to humans. Click here for file

Additional File 5 Playback of the S20 motif in Figure 1e with the time scale expanded by a factor of 10 to render the fundamental frequency audible to humans. Click here for file

Additional File 6 Playback of the L20 motif in Figure 1f with the time scale expanded by a factor of 10 to render the fundamental frequency audible to humans. Click here for file

Additional File 7 Playback of the BARK motif in Figure 1g with the time scale expanded by a factor of 10 to render the fundamental frequency audible to humans. Click here for file

Additional File 8 Playback of shrew (*Sorex trowbridgei*) echolocation recorded on Upper (spectrogram not shown) with time expanded by a factor of 10 to render the fundamental frequency audible to humans. Click here for file

Additional File 9 Playback of bat echolocation recorded on Upper (spectrogram not shown) with time expanded by a factor of 10 to render the fundamental frequency audible to humans. Note the "feeding buzz" as the bat approaches and captures insect prey. Click here for file

Additional File 10 Descriptive statistics for components of each of the 7 motifs. Click here for file
