# Supplementary material for: Production of ultrasonic vocalizations by Peromyscus mice in the wild
Source: Front Zool. 2006 Feb 28;3:3. doi: 10.1186/1742-9994-3-3 (PMC1524959; doi:10.1186/1742-9994-3-3)
Supplement: Additional File 10 — Descriptive statistics for components of each of the 7 motifs. [file 1742-9994-3-3-S10.pdf]

Summary Table for the 2 Part Whistle (2PW) Motif. Data are presented as means±1SE with ranges in brackets.

n=8 phrases	First syllable	Second syllable
Duration (msec)	201.83±13.46 (151.98-251.00)	122.48±8.96 (75.99-161.85)
starting F (kHz)	24.26±2.00 (15.03-31.74)	31.76±1.81 (22.38-39.09)
ending F (kHz)	21. 78±1.05 (15.7-24.7)	28.99±0.94 (24.72-31.40)
high F (kHz)	25.90±0.82 (21.4-31.7)	32.84±1.24 (29.06-39.09)
low F (kHz)	20.15±1.27 (15.0-24.38)	27.90±1.20 (22.38-31.40)
bandwidth (kHz)	5.74±0.82 (3.34-9.69)	5.03±0.69 (0.33-13.70)
F at maximum amplitude (kHz)	24.83±1.30 (19.4-30.6)	26.44±0.60 (24.57-29.72)
Slope (kHz/msec)	0.06±0.03 (0.02-0.23)	0.10±0.04 (0.03-0.41)
Duration between the two syllables	89.97±11.13 (33.04-138.77)	

Summary Table for the 3 Part Whistle (3PW) Motif. Data are presented as means±1SE with ranges in brackets.

n=18 phrases	First syllable	Second syllable	Third syllable
Duration (msec)	157.12±9.30 (102.20-238.99)	161.55±8.82 (114.98-240.09)	139.58±10.31 (79.74-235.68)
starting F (kHz)	21.19±1.44 (12.28-33.74)	23.54±1.63 (13.26-33.19)	24.24±1.66 (14.24-34.74)
ending F (kHz)	19.15±1.15 (11.79-28.40)	23.20±1.75 (12.77-32.07)	28.24±2.88 (12.77-34.74)
high F (kHz)	23.48±1.74 (12.28-37.08)	28.03±2.30 (14.73-42.09)	27.77±2.09 (15.72-39.09)
low F (kHz)	18.70±1.06 (11.79-28.40)	22.34±1.64 (12.77-32.07)	23.23±1.75 (12.77-34.74)
bandwidth (kHz)	2.95±0.64 (0.46-10.02)	2.05±0.38 (0.00-5.35)	1.87±0.39 (0.00-6.01)
F at maximum amplitude (kHz)	21.40±1.45 (13.35-36.64)	24.35±0.38 (13.35-33.19)	26.24±2.09 (14.21-34.91)
Slope (kHz/msec)	0.07±0.02 (0.003-0.27)	0.13±0.04 (0.01-0.46)	0.10±0.03 (0.01-0.45)
Duration between the first and second syllable	123.59±10.01 (70.49-198.24)		
Duration between the second and third syllable	130.81±10.82 (77.09-226.87)		

Summary Table for the 4 Part Whistle (4PW) Motif. Data are presented as means±1SE with ranges in brackets.

n=13 phrases	First syllable n=13	Second syllable n=13	Third syllable n=13	Fourth syllable* n=10
Duration (msec)	85.74±9.36 (26.65-123.35)	172.97±7.94 (107.95-216.87)	144.40±6.49 (104.63-182.82)	65.55±8.04 (40.08-117.84)
starting F (kHz)	21.71±2.12 (15.22-45.10)	25.58±1.31 (20.67-35.77)	24.78±1.53 (19.38-38.09)	28.15±1.68 (23.28-36.75)
ending F (kHz)	17.24±0.65 (13.26-21.11)	21.87±1.21 (15.72-29.47)	23.28±1.37 (18.17-30.45)	25.65±1.81 (20.05-33.74)
high F (kHz)	21.71±2.12 (15.22-45.10)	26.71±1.57 (20.67-37.75)	27.34±1.98 (19.38-38.09)	28.45±1.75 (23.28-36.75)
low F (kHz)	17.24±0.65 (13.26-21.11)	21.64±1.12 (15.72-29.47)	22.71±1.21 (18.17-30.45)	25.59±1.79 (20.04-33.74)
bandwidth (kHz)	4.47±1.80 (1.00-25.27)	4.17±0.98 (0.05-14.40)	2.65±0.96 (0.00-13.70)	2.6±0.48 (0.49-5.68)
F at maximum amplitude (kHz)	19.69±1.33 (14.64-33.62)	25.02±1.47 (18.53-33.62)	24.95±1.60 (18.53-35.75)	26.72±2.11 (19.40-35.75)
Slope (kHz/msec)	0.07±0.04 (0.01-0.60)	0.03±0.01 (0.00-0.15)	0.07±0.03 (0.00-0.40)	0.07±0.01 (0.01-0.16)
Duration between the first and second syllable	125.55±12.30 (50.66-182.82)			
Duration between the second and third syllable	139.95±17.04 (59.47-213.66)			
Duration between the third and fourth syllable	147.04±12.81 (83.70-242.29)			

*3 syllables cut off by tape and not included in the analysis

Summary Table for the Short 20 (S20) Motif. Data are presented as means±1SE with ranges in brackets.

n=8 phrases*	First syllable n=8	Second syllable n=7
Duration (msec)	28.44±3.24 (16.39-42.84)	35.83±7.12 (14.60-69.75)
high F (kHz)	20.56±1.02 (14.24-23.26)	21.25±0.64 (18.66-23.26)
low F (kHz)	19.78±0.93 (14.24-22.40)	20.54±0.63 (18.17-22.59)
bandwidth (kHz)	0.78±0.31 (0.49-2.45)	0.71±0.20 (0-1.47)
F at maximum amplitude (kHz)	20.19±0.83 (15.07-22.40)	20.99±0.44 (19.38-22.40)
Slope (kHz/msec)	0.04±0.01 (0.00-0.09)	0.03±0.01 (0.00-0.10)
Duration between the first and second syllable (n=7)	357.93±25.16 (319.38-506.61)	
Duration between the second and third syllable (n=6)	436.49±45.54 (317.18-654.19)	

*The number of syllables in each phrase was 1-4. The first two syllables (where possible) were selected from each phrase and the first and second intervals (where possible) were selected from each phrase.

Summary Table for the Frequency Modulated Short20 (FMS20) Motif. Data are presented as means±1SE with ranges in brackets.

n=5 phrases*	Second syllable	Third syllable
Duration (msec)	22.54±5.06 (11.34-40.42)	38.58±6.28 (16.17-53.74)
high F (kHz)	33.66±7.62 (18.17-58.79)	45.68±5.97 (24.72-60.78)
low F (kHz)	22.63±2.56 (16.70-29.96)	22.44±1.94 (17.19-28.86)
bandwidth (kHz)	11.03±6.81 (1.47-36.75)	23.24±6.81 (1.34-38.06)
F at maximum amplitude (kHz)	23.27±1.64 (18.95-28.42)	22.41±1.83 (17.23-28.42)
Slope (kHz/msec)	0.47±0.24 (0.08-1.17)	0.80±0.31 (0.03-1.84)
Duration between the first and second syllable	352.86±26.74 (312.78-451.54)	
Duration between the second and third syllable	370.48±30.77 (286.34-455.94)	

*The number of syllables in each phrase was 4. The two middle syllables were selected from each phrase and the first and second intervals were selected from each phrase.

Summary Table for the Long 20 (L20) Motif. Data are presented as means±1SE with ranges in brackets.

n=9 phrases*	First syllable n=9	Second syllable n=7
Duration (msec)	188.26±23.81 (58.44-262.12)	165.26±23.49 (42.29-222.47)
high F of fundamental (kHz)	20.58±0.41 (18.17-22.85)	21.00±0.35 (19.66-22.72)
low F of fundamental (kHz)	20.02±0.44 (15.7-24.7)	20.57±0.36 (19.64-22.38)
bandwidth (kHz)	0.56±0.08 (0.33-0.98)	0.42±0.15 (0.00-0.98)
F at maximum amplitude (kHz)	21.20±0.25 (19.4-31.0)	20.61±0.81 (15.95-21.96)
Slope (kHz/msec)	0.003±0.00 (0.001-0.008)	0.003±0.001 (0.00-0.008)
Duration between the first and second syllable (n=9)	207.00±18.76 (134.36-321.59)	
Duration between the second and third syllable (n=5)	248.69±17.27 (213.66-299.56)	

*The number of syllables in a phrase varied from 2-4; There were 9 phrases total. The first two syllables was selected from each phrase and the first and second (where possible) intervals were selected from each phrase.

Summary Table for the BARK Motif. Data are presented as means±1SE with ranges in brackets.

n=4 phrases*	
Duration (msec)	18.83±2.1<br>(15.64-24.32)
starting F (kHz)	16.97±3.15<br>(11.7-26.1)
ending F (kHz)	14.88±0.86<br>(12.7-16.7)
high F (kHz)	19.55±2.24<br>(16.4-26.1)
low F (kHz)	13.63±1.78<br>(11.7-15.7)
bandwidth (kHz)	4.6±2.99<br>(0.00-13.4)
F at maximum amplitude (kHz)	18.43±2.83<br>(16.4-22.4)
Slope (kHz/msec)	0.28±0.39<br>(0.00-0.85)
Duration between the two syllables	143.33±18.56<br>(120.00-180.00)

*The number of syllables varied from 1-4; There were 4 phrases total. A single syllable was selected from each phrase and a single interval selected from each phrase.
